# Habitat use of the ocelot (*Leopardus pardalis*) in Brazilian Amazon

**DOI:** 10.1002/ece3.5005

**Published:** 2019-04-19

**Authors:** Bingxin Wang, Daniel G. Rocha, Mark I. Abrahams, André P. Antunes, Hugo C. M. Costa, André Luis Sousa Gonçalves, Wilson Roberto Spironello, Milton José de Paula, Carlos A. Peres, Juarez Pezzuti, Emiliano Ramalho, Marcelo Lima Reis, Elildo Carvalho Jr, Fabio Rohe, David W. Macdonald, Cedric Kai Wei Tan

**Affiliations:** ^1^ Wildlife Conservation Research Unit Department of Zoology The Recanati‐Kaplan Centre University of Oxford Tubney, Oxon UK; ^2^ State Key Laboratory of Vegetation and Environmental Change Institute of Botany The Chinese Academy of Sciences Beijing China; ^3^ University of Chinese Academy of Sciences Beijing China; ^4^ Graduate Group in Ecology Department of Wildlife, Fish, and Conservation Biology University of California Davis Davis California; ^5^ Grupo de Ecologia e Conservação de Felinos na Amazônia Instituto de Desenvolvimento Sustentável Mamirauá Tefé Brazil; ^6^ Field Conservation and Science Department Bristol Zoological Society Bristol UK; ^7^ Redefauna - Rede de Pesquisa em Biodiversidade Conservação e Uso da Fauna da Amazônia Manaus Brazil; ^8^ Programa de Pós‐graduação em Ecologia e Conservação da Biodiversidade Universidade Estadual de Santa Cruz Ilhéus Brazil; ^9^ Grupo de Pesquisa de Mamíferos Amazônicos Instituto Nacional de Pesquisas da Amazônia Manaus Brazil; ^10^ Centre for Advanced Amazon Studies University of Para Altamira Brazil; ^11^ Programa de Pós-Graduação em Ecologia Universidade Federal do Pará e EMBRAPA Amazônia Oriental Belém Brazil; ^12^ School of Environmental Science Cetre for Ecology, Evolution and Conservation University of East Anglia Norwich UK; ^13^ Instituto de Desenvolvimento Sustentável Mamirauá Tefé Brazil; ^14^ ICMBio/CNPq Brasília Brazil; ^15^ Centro Nacional de Pesquisa e Conservação de Mamíferos Carnívoros Instituto Chico Mendes de Conservação da Biodiversidade Atibaia Brazil; ^16^ Faculty of Ecology and Natural Resource Management Norwegian University of Life Sciences Ås Norway; ^17^ Programa de Pós‐graduação em Genética, Conservação e Biologia Evolutiva – GCBEv Instituto Nacional de Pesquisas da Amazônia – INPA Manaus Brazil; ^18^ Wildlife Conservation Society Brazil – Amazon Program Manaus Brazil

**Keywords:** Brazilian Amazon, camera traps, mesopredator, occupancy, ocelot, restricted spatial regression

## Abstract

Amazonia forest plays a major role in providing ecosystem services for human and sanctuaries for wildlife. However, ongoing deforestation and habitat fragmentation in the Brazilian Amazon has threatened both. The ocelot is an ecologically important mesopredator and a potential conservation ambassador species, yet there are no previous studies on its habitat preference and spatial patterns in this biome. From 2010 to 2017, twelve sites were surveyed, totaling 899 camera trap stations, the largest known dataset for this species. Using occupancy modeling incorporating spatial autocorrelation, we assessed habitat use for ocelot populations across the Brazilian Amazon. Our results revealed a positive sigmoidal correlation between remote‐sensing derived metrics of forest cover, disjunct core area density, elevation, distance to roads, distance to settlements and habitat use, and that habitat use by ocelots was negatively associated with slope and distance to river/lake. These findings shed light on the regional scale habitat use of ocelots and indicate important species–habitat relationships, thus providing valuable information for conservation management and land‐use planning.

## 1 INTRODUCTION

South America's Amazon basin harbors over half of all the tropical rainforests left on Earth, spanning a vast area of 6.7 million km^2^ (Wittmann & Junk, 2016), and is home to roughly half of the world's species (Shukla, Nobre, & Sellers, 1990). Unfortunately, human‐induced changes to its ecosystem, for a host of social‐economic reasons, are causing widespread biodiversity declines in the Amazon (Gibson et al., 2011; Newbold et al., 2015; Guilherme de Andrade Vasconcelos, 2017). Over 2000–2012, the average rate of tropical dense forests loss was 74,400 km^2^/year (Malhi, Gardner, Goldsmith, Silman, & Zelazowski, 2014). Deforestation is intensifying pressures on forest vertebrates, as well as on indigenous and nonindigenous forest dwellers and their livelihoods. In addition, the process of deforestation is not random, with remaining forests often being confined to steep slopes and hilltops unsuitable for both large‐scale agriculture and cattle ranch. This leads to habitat fragmentation and population isolation (Malhi et al., 2014), especially throughout the so‐called arc of deforestation region, which together influence the nature and frequency of species interactions with unknown cascading effects on long‐term biodiversity persistence (Haddad et al., 2015).

Forest carnivores, especially apex predators, are thought to be particularly vulnerable and sensitive to deforestation and forest fragmentation (Noss, Quigley, Hornocker, Merrill, & Paquet, 1996) because of their restricted carnivorous diet (Vetter, Hansbauer, Végvári, & Storch, 2011) and large home ranges. They are essential for maintaining the community structure within a foodweb, and are vital to ecosystem functioning (Ripple et al., 2014). Mesopredators can fill this role to some degree when apex predators are eradicated or depleted (Prugh et al., 2009). Some omnivorous mesopredators, typically opportunists with broad diets, such as raccoons (*Procyon lotor*), may respond positively to anthropogenic resources with behavioral change (Prange & Gehrt, 2004). In these cases, mesopredators with good adaptability might serve as a buffer to sustain ecosystem stability and integrity when apex predators are inadequate. Alternatively, mesopredators are sometimes associated with unpredictable cascade effects, such as disease outbreaks and human–wildlife conflicts (Prugh et al., 2009). These various, and unpredictable, possibilities provide a background for an interest in medium‐sized Neotropical cats in addition to the fundamental interest in their poorly documented autecology.

**Figure 1:**
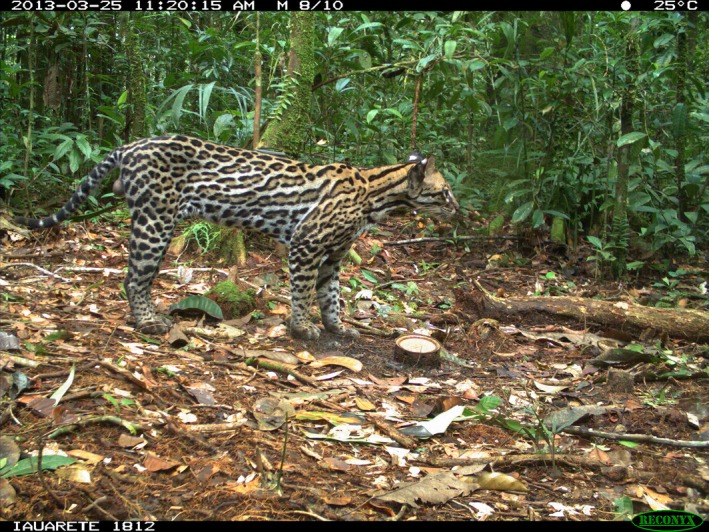
Ocelot was taken by one camera trap in 2013 (photos provided by Daniel G. Rocha)

The ocelot *Leopardus pardalis* (Linnaeus, 1758; Figure 1) is a medium‐sized (6.6–18.6 kg) Neotropical spotted cat with a broad geographic distribution in the Americas, ranging from the extreme south of Texas (USA), throughout Mesoamerica and the Amazon, to open environments in northern Argentina and flood plains, dry coniferous forests, and rainforests (Emmons & Feer, 1998; Murray & Gardner, 1997). Ocelots are considered solitary, nocturnal/crepuscular, and semi‐arboreal and are excellent climbers (Di Bitetti, Paviolo, & De Angelo, 2006). Documented home ranges are average 12.5 ± *SE* 3.4 km^2^ (Gonzalez‐Borrajo, López‐Bao, & Palomares, 2016). They have been recorded at elevations up to 1,200 m (Nowell & Jackson, 1996) and are classified as Least Concern on the IUCN Red List (Paviolo et al., 2015). They were heavily exploited in Amazonia by the international fur trade between the 1930s and mid‐1970s (Antunes et al., 2016; Smith, 1976). Currently, ocelots suffer habitat loss, fragmentation, and other anthropogenic pressures, such as oil exploration (Kolowski & Alonso, 2010), vehicle collisions, illegal trade, and retaliatory killing due to depredation on small livestock (Paviolo et al., 2015).

Nevertheless, ocelot, a mesopredator, has been studied much less than larger, more charismatic, felids, such as jaguar (*Panthera onca*) and puma (*Puma concolor*). Since 2000, studies of ocelot using camera traps have proliferated (Blake et al., 2015; de Oliveira et al., 2010; Paviolo et al., 2015; Pratas‐Santiago, Gonçalves, da Maia Soares, & Spironello, 2016; Wang, 2002), in particular, those estimating the species’ abundance and density (Di Bitetti, Paviolo, De Angelo, & Di Blanco, 2008; Di Bitetti et al., 2006; Dillon & Kelly, 2007; Penido et al., 2016; Rocha, Sollmann, Ramalho, Ilha, & Tan, 2016). These studies have revealed various aspects of ocelot ecology (Supporting Information Table S1), and three of them used the occupancy modeling framework: two of them investigated the interactions between ocelots and sympatric species (Massara, Paschoal, Bailey, Doherty, & Chiarello, 2016; Massara, de Oliveira Paschoal et al., 2018; Massara, Paschoal et al., 2018), the third investigated how an attractant affected detection (Cove, Spinola, Jackson, & Saenz, 2014). Other studies report that ocelot densities correlate with forest cover (Paviolo et al., 2015), precipitation (Maffei, Noss, Cuéllar, & Rumiz, 2005; Rocha et al., 2016), and latitude (Di Bitetti et al., 2008; Rocha et al., 2016); in addition, ocelots may have an affinity for some specific matrices, such as eucalyptus plantation (Massara, de Oliveira Paschoal et al., 2018; Massara, Paschoal et al., 2018). Ocelots have been recorded in a great variety of habitats, from heavily logged and fragmented forests, to early and late successional forests, the outskirts of major cities and towns, disturbed scrub/woodland Savannah and agricultural areas (de Oliveira et al., 2010). Notwithstanding these fragments of research, studies on the habitat preference of ocelots on a regional scale are lacking.

Occupancy modeling has become a popular tool for investigating species occurrence over temporal and spatial scales. This type of model estimates the probability of a site being occupied by a species, taking into account imperfect detection processes (Mackenzie et al., 2002).

We use camera trap detection/nondetection data from 12 sites in Brazilian Amazonia to examine the habitat use of the ocelot. This is by far the largest known dataset for this species. Our key objective is to reveal the influence of different environmental variables and anthropogenic impacts on ocelot occupancy at a landscape scale and thus predict its habitat use across the Brazilian Amazon.

## 2 METHODS

### 2.1 Study area

Data were collected across twelve sites in the Amazon basin, Brazil from 2010 to 2017: (a) Cabo Frio and Km 37 experimental forest reserves, from part of the Biological and Dynamics of Forest Fragments Project (PBDFF) (Laurance, Ferreira, Rankin‐de Merona, & Laurance, 1998), (b) Cuieiras Forest Reserve and Tropical Forestry Experimental Station (ZF2), (c) Adolpho Ducke Forest Reserve (DUCKE), (d) Amanã Sustainable Development Reserve (RDSA), (e) Médio Juruá Extractive Reserve and Uacarí Sustainable Development Reserve (REMJ & RSUA), (f) Uatumã Biological Reserve (Uatuma), (g) Campos Amazônicos National Park (PNCA), (h) Mapinguari National Park (PNM), (i) Juruena National Park (PNJU), (j) Terra do Meio Ecological Station (TMES), (k) São Benedito River (SBR), (l) Nascentes do Lago Jari National Park, Igapó‐Açu Sustainable Development Reserve and Tupana Settlement Project (BRA319). Apart from the São Benedito River (Serra do Cachimbo), which is a private area, and the Tupana Settlement Project, the sites are located in protected areas or reserves. The climatic classification of this region, according to Köppen (Kottek, Grieser, Beck, Rudolf, & Rubel, 2006), is tropical moist climate. The entire survey region consisted of a similar baseline mosaic of tropical forest, mostly upland nonfloodable *terra firme* forests (dry land/solid ground) and, to a lesser extent, seasonally flooded forests.

### 2.2 Camera trap survey

**Figure 2:**
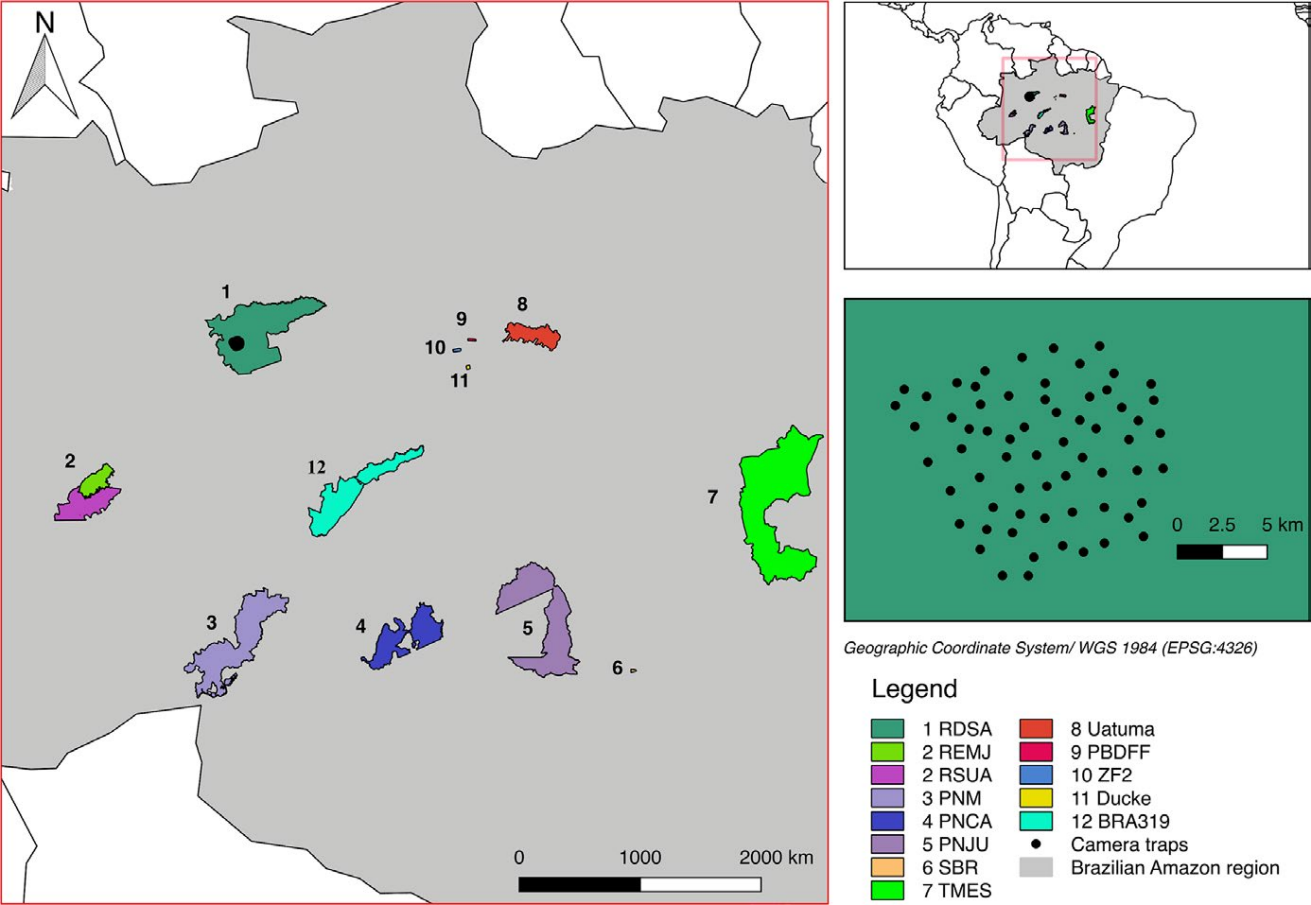
Map with the camera trap surveyed areas used to model ocelot habitat use in Central Amazon, Brazil. Protected areas: *Amanã Sustainable Development Reserve* (RDSA); *Médio Juruá Extractive Reserve* and *Uacarí Sustainable Development Reserve* (REMJ & RSUA); *Campos Amazônicos National Park* (PNCA); *Mapinguari National Park* (PNM); *Adolpho Ducke Forest Reserve* (DUCKE); *Cabo Frio and Km 37 experimental forest reserves* (PBDFF); *Cuieiras Forest Reserve and Tropical Forestry Experimental Station* (ZF2); *The Juruena National Park* (PNJU); *Terra do Meio Ecological Station* (TMES); *São Benedito River* (SBR); *Uatumã* (Uatuma); *Nasentes do Lago Jari National Park and IGAP‐AU Sustainable Development* (BRA319). Projection: WGS84, Datum: WGS 1984 (EPSG4326)

Data collection and surveys at most of our study areas were designed to study large mammals like jaguars, so our data on ocelots represent by‐catch (except for the REMJ and RSUA dataset, see methods in Costa, Peres, & Abrahams, 2018). In Malaysia, Tan et al. (2017) used them to estimate habitat use of clouded leopards, as did Penjor, Macdonald, Wangchuk, Tandin, and Tan (2018) in Bhutan. Camera trapping, although originally motivated by studies of large mammals, yielded data on ocelots (Figure 2). In total, 899 unbaited camera trap stations were operated, involving a total survey effort of 40,347 days, yielding 334 independent detections of ocelots. The independent detection events were defined as the consecutive conspecific images with >30 min apart at the same camera trap station. Stations at RDSA had two cameras facing each other 4–5 m apart and stations at all other survey areas had only single cameras. All camera trap stations were placed at approximately 30–50 cm above ground along randomly selected transects in different surveyed sites, perpendicular to existing trails or animal tracks used for previous censuses of primates and terrestrial vertebrates to enhance the opportunity to detect the focal species (Di Bitetti et al., 2006). The sensitivity sensor was set at “high.” Camera traps were operational for 24 hr a day during the monitoring period, aside from malfunctions, damage, or theft. Details of camera trap deployment (the numbers of stations, effort, mean trap spacing, and total numbers of records of ocelot) are provided in Table 1.

**Table 1:** Details of camera trap survey for ocelots in Brazilian Amazon

Year	Site	Area (km^2^)	Stations	Effort	No. of camera traps per station	Spacing (*SD*) in m	Records of ocelots
2010	PDBFF (Manaus)	350	30	946	1	1,365.08 (71.90)	10
2010	ZF2 (Manaus)	380	30	1,050	1	1,389.33 (19.32)	8
2010–2011	BRA319	8,127.4518	196	9,647	1	312.79 (321.94)	8
2012	DUCKE (Manaus)	100	30	1,877	1	1,351.25 (87.99)	4
2013–2014	RDSA	23,500	64	2,682	2	1,245.76 (262.50)	45
2013–2014	REMJ & RSUA	886.22	183	6,169	1	457.70 (265.84)	48
2014	Uatuma	1,601.704	95	2,867	1	1,153.32 (1055.38)	5
2015	REMJ & RSUA	886.22	25	1,112	1	7,371.60 (4367.87)	14
2016	PNCA	9,613	86	5,537	1	2,872.18 (1048.53)	28
2016	PNM	17,228.52	58	1,939	1	3,747.17 (1813.93)	57
2016	PNJU	19,582.03	18	1,276	1	987.64 (13.28)	16
2016	TMES	3,373.111	61	3,652	1	1,340.78 (60.59)	86
2017	SBR	8.31	23	1,593	1	1,380.649 (135.88)	5
	Total		899	40,347			334

*Notes*

Effort is in number of camera trap × days, the spacing is the average distance between camera traps and their nearest neighbor.

### 2.3 Data analysis

Detection histories based on photographic records were constructed in a two dimensional matrix format. Data were analyzed using (a) single‐species, single‐season occupancy models in a maximum‐likelihood framework (Mackenzie et al., 2002), which can help to select the most informative covariates, and (b) single‐season spatial occupancy models that account for spatial autocorrelation in a Bayesian framework (Johnson, Conn, Hooten, Ray, & Pond, 2013). The latter method was used as our study combined multiple protected areas at varying distances apart, distributed across the Brazilian Amazon basin. To minimize the possibility of violating the assumption of population closure (Rota, Fletcher, Dorazio, & Betts, 2009), only the first 120‐day period of each survey was included in the analysis. Collapsing sampling periods minimizes the failure of convergence in models when overall detection probability is low (Dillon & Kelly, 2007; Otis, Burnham, White, & Anderson, 1978). It can also increase temporal independence among occasions (Dillon & Kelly, 2007). The 120‐day data subsets were collapsed into multiple‐day sampling occasions (7, 10, 12, 15 days of period) to maximize temporal independence of captures. The optimum number of days per occasion was selected based on a chi‐square goodness‐of‐fit (MacKenzie & Bailey, 2004) test for the global model performed with 1,000 bootstraps. A 12‐day period represented the optimum number of days to maximize model fit (Supporting Information Table S2).

Building on previous studies of similar mesopredators, such as golden cats (*Pardofelis teminckii*) and clouded leopards (*Neofelis nebulosa*; Haidir, Dinata, Linkie, & Macdonald, 2013; Tan et al., 2017), we interpreted ocelot occupancy as a proxy for habitat use of ocelot. Habitat use was modeled by occupancy models using three types of covariates: (a) habitat use covariates on natural environment: elevation, slope (mean angle of slope), forest cover(VCF, GFC30, GFC50, GFC75, GFC90), distance to rivers and distance to lakes, (b) habitat use covariates on human activity and fragmentation:, distance to roads, distance to settlements, and measures of forest fragmentation (CWED, Contig, DCAD), and (c) detection covariates that describe each of surveyed sites: survey site (the 12 different surveyed sites) and effort (number of days that each camera trap station was active within occasions). The summary statistics of each of these covariates are tabulated (Supporting Information Table S3). We hypothesized that ocelots would have a bias for flat land, dense forests, areas near rivers/lakes and avoid approaching roads, settlements and fragmented forests. For the detection covariates, we hypothesized that the higher the camera trapping effort, the higher probability of detecting focal species. Different surveyed sites would have different detection probabilities due to geographical and biological features. The occupancy covariates at each camera trap location were generated using QGIS version 2.18.9 (QGIS Development Team, 2017). Elevation and slope values were extracted from a 30 × 30 m of resolution digital elevation model (DEM), the Shuttle Radar Topography Mission (USGS, 2003), downloaded from *U.S. Geological Survey* (https://earthexplorer.usgs.gov/). The distance to rivers/lakes and paved roads was produced using Cartographic Integrated Basis Digital CIM IBGE (IBGE, 2011). The distance to settlements was from an open source (OpenStreetMap Contributors, 2015, https://planet.openstreetmap.org), including towns, villages, and isolated settlements. Vegetation Continuous Forest of 250‐m resolution (DiMiceli et al., 2011) and 30‐m resolution Global Forest Change (Hansen et al., 2013) was used as measures of forest cover. Specifically, the Global Forest Change layer (Hansen et al., 2013) allows users to set a threshold of percentage of tree cover that is to be considered as forest for the area of interest. On account of this and a previous similar study (Tan et al., 2017), we set four different threshold values (30%, 50%, 75% and 90%). Forest fragmentation variables such as CWED (Contrast‐weighted edge density is a measure of edge density standardized to a per unit area), Contig (Contiguity index is an index of spatial connectedness of forest), and DCAD (Disjunct core area density is the number of disconnected patches of suitable interior habitat per unit area) were chosen to examine the effects of edge and forest fragmentation on ocelot habitat use. The measures of forest fragmentation dataset were produced by FRAGSTATS 4 (McGarigal, Cushman, Neel, & Ene, 2002). For all above continuous covariates, values were extracted from the mean of all raster cells included in a 500‐m radius around each camera trap station and were derived using the “zonal statistics” tool in QGIS. This radius was chosen to represent an overview of the environmental setting and habitat type surrounding each camera trap station. Due to the limited availability of VCF and GFC maps (the latest maps are for years 2010 and 2014, respectively), we used the temporally closest one.

Statistical analyses were undertaken in two parts. The first selected the most informative covariates. First, Pearson's correlation test was conducted to examine collinearity between continuous covariates. Covariates with *r* > |0.6| were considered correlated. Second, univariate occupancy models were conducted with R package “unmarked” (Fiske & Rochard, 2011) and we selected the covariate (of the correlated pair) based on the model with lower ΔAIC value. We used the “AICcmodavg” package (Mazerolle, 2017) in R (R Development Core Team, 2017) for this second step. In order to avoid bias from correlated detections due to spatial replicates that are not sampled randomly, we conducted occupancy models in program PRESENCE (Hines, 2006) account for correlated detections (Hines et al., 2010) to checking for the effect of correlated detections (Supporting Information Table S6). Third, the best candidate model including the most informative covariates was selected by AIC_c_ (corrected Akaike's information criterion, used due to small‐sample correction). Models with all possible combinations of remaining covariates were compared, and the models within ΔAIC_c_ < 2 were considered to the best‐performing models (Burnham & Anderson, 2004). The dredging command in the multi‐model inference package “MuMIn” (Bartoń, 2013) was used to average the parameters in R (Team RC, 2017). Finally, based on the summed model weights (importance; Barbieri & Berger, 2004; Kalies, Dickson, Chambers, & Covington, 2012), the most influential covariates (importance > 0.5) were retained for the subsequence analysis.

The second part of the statistical process used the R package “stocc” to account for spatial autocorrelation (Johnson, 2015). A restricted spatial regression model (RSR) was used to generate the spatial autocorrelation parameter. RSR models use an efficient Gibbs sampler Markov chain Monte Carlo method to make Bayesian inference about the detection and occupancy processes and models were fitted using a probit link function (probit link, uses the inverse of the cumulative distribution function of the standard normal distribution to transform probabilities to the standard normal variable, Razzaghi, 2013) instead of the logit link function used in the first part. This increased computational efficiency (Johnson et al., 2013). In the RSR model, the threshold was set to 1.99 km according to the average ocelot home range (12.46 ± *SE* 3.39 km^2^, which corresponded to 1.99 km radius; Gonzalez‐Borrajo et al., 2016) and moran.cut 89.9 (0.1*number of camera trap stations), as recommended by Hughes and Haran (2013). For each Bayesian model, the Gibbs sampler was run for 50,000 iterations following a burn‐in of 10,000 iterations that were discarded, and a thinning rate of 5 (Tan et al., 2017). We applied an improved occupancy‐based modeling approach that incorporates spatial autocorrelation. This improved model included a spatial component which can help to mitigate bias from nonindependent environmental covariates (Johnson et al., 2013). All statistical analyses for this study were conducted in the R software environment v.3.3.3 (R Development Core Team, 2017).

## 3 RESULTS

### 3.1 Selection of contributing covariates

#### 3.1.1 Detection covariates

Both site and effort strongly contributed to variation in the detection probability of ocelot. PNM had the highest detection probability, followed by TMES, PNJU, and RDSA. PNCA had the lowest detection probability (Table 3). Effort was positively correlated to detection probability (beta = 0.175, *SE* = 0.029, Table 3).

#### 3.1.2 Occupancy covariates

There was correlation among all forest cover covariates (VCF and GFC30, 50, 75, 90) and among all measures of forest fragmentation (CWED, Contig and DCAD). Based on these correlations and the performance of each covariate in the univariate habitat use models (Supporting Information Table S4), GFC30, D.ROA, D.RIV, D.LAK, D.SET, ELE, SLO, and DCAD were selected for the further analysis.

### 3.2 Selection of the best model

**Figure 3:**
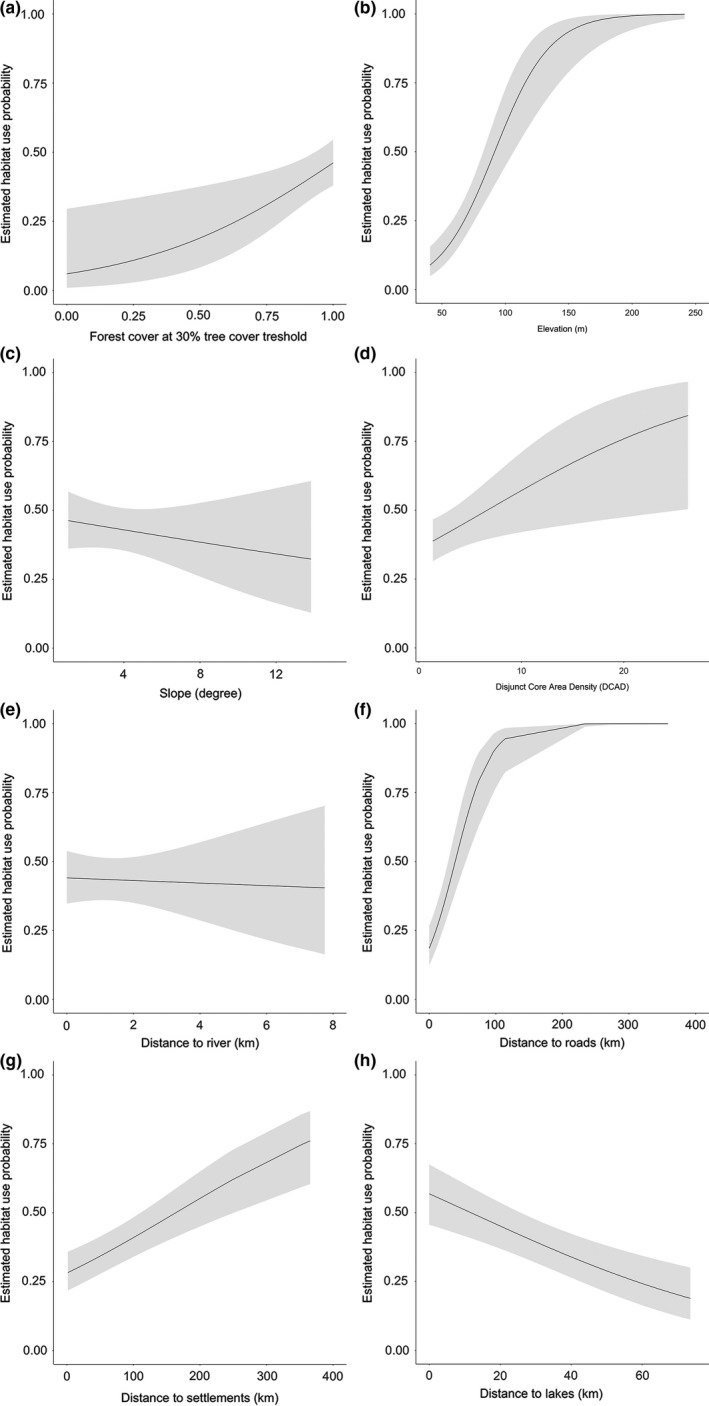
Relationship between ocelot estimated habitat use probability and occupancy covariates with summed model weights >0.3. (a) Global Forest Change Threshold 30%; (b) elevation; (c) slope; (d) disjunct core area density; (e) distance to river; (f) distance to roads; (g) distance to settlements; (h) distance to lakes

Among the models that incorporated all possible combinations of the eight occupancy covariates, sixteen models (out of 256) had ΔAIC < 2 from the top ranked model (Table 2). The best candidate model was *p*(site + effort), *ψ*[forest cover (GFC30)] with a highest weight of 0.11. Based on the summed model weight (importance), all of the covariates had some degree of influence on the habitat use of ocelot (importance from 0.3 to 1; Table 3). Specifically, habitat use by ocelot was strongly positively associated with forest cover (GFC30; importance = 1.0; Table 3; Figure 3a), with DCAD (importance = 0.51; Table 3; Figure 3d) and strongly negatively related to slope (SLO; importance = 0.58; Table 3; Figure 3c). There was a weaker positive sigmoidal correlation between habitat use and distance to roads, which then leveled off at higher values of distance to roads (D.ROA; importance = 0.46; Table 3; Figure 3f) and there was a weaker negative relationship between habitat use and distance to river (D.RIV; importance = 0.42; Table 3; Figure 3e). The rest of covariates had importance <0.4 (see details in Table 3 and Figure 3) Our results indicated that the covariates forest cover (GFC30), slope (SLO) and disjunct core area density (DCAD) attained a summed model weight (importance) of >0.5 (Table 3), which were used in the subsequent phase to test for spatial autocorrelation.

**Table 2:** Multivariate model selection results of ocelot with AIC_c_ < 2

Model	AIC_c_	ΔAIC_c_	AIC_c_wt	*K*	Log likelihood
*ψ*(GFC30), *p*(site + effort)	1,767.78	0	0.11	15	−868.62
*ψ*(GFC30 + D.ROA + D.LAK + DCAD + ELE + SLO), *p*(site + effort)	1,768.09	0.32	0.09	20	−863.57
*ψ*(GFC30 + SLO), *p*(site + effort)	1,768.18	0.41	0.09	16	−867.78
*ψ*(GFC30 + D.ROA + DCAD + ELE + SLO), *p*(site + effort)	1,768.25	0.48	0.08	19	−864.69
*ψ*(GFC30 + D.ROA + DCAD + SLO), *p*(site + effort)	1,768.52	0.75	0.07	18	−865.87
*ψ*(GFC30 + D.RIV + SLO), *p*(site + effort)	1,768.8	1.02	0.06	17	−867.05
*ψ*(GFC30 + DCAD), *p*(site + effort)	1,768.85	1.08	0.06	16	−868.12
*ψ*(GFC30 + DCAD + SLO), *p*(site + effort)	1,768.91	1.13	0.06	17	−867.11
*ψ*(GFC30 + D.RIV + D.ROA + DCAD + SLO), *p*(site+effort)	1,769.04	1.26	0.06	19	−865.09
*ψ*(GFC30 + D.RIV + DCAD), *p*(site + effort)	1,769.23	1.45	0.05	17	−867.27
*ψ*(GFC30 + D.RIV + D.LAK), *p*(site + effort)	1,769.32	1.54	0.05	17	−867.31
*ψ*(GFC30 + D.RIV + DCAD + SLO), *p*(site + effort)	1,769.34	1.56	0.05	18	−866.28
*ψ*(GFC30 + D.LAK), *p*(site + effort)	1,769.55	1.77	0.04	16	−868.47
*ψ*(GFC30 + D.SET), *p*(site + effort)	1,769.66	1.88	0.04	16	−868.52
*ψ*(GFC30 + D.RIV + D.ROA + D.LAK + DCAD + ELE + SLO), *p*(site + effort)	1,769.71	1.94	0.04	21	−863.33
*ψ*(GFC30 + D.ROA + SLO), *p*(site + effort)	1,769.74	1.96	0.04	17	−867.52

*Notes*

AIC_c_ Akaike's information criterion corrected for finite sample sizes. ΔAIC_c_ relative difference in AIC_c_ values compared with the top ranked model, AIC_c_wt weight, *K* number of parameters. Site covariates tested were: elevation (ELE), slope (SLO), distance to river (D.RIV), distance to lakes (D.LAK), distance to roads (D.ROA), distance to settlements (D.SET), Global Forest Change with threshold values 30 (GFC30) and disjunct core area density (DCAD). Detection covariates tested were: effort and site.

**Table 3:** Summed model weights for covariates used to model the probabilities of occupancy and detection of ocelots

Covariate	Summed model weights	*β*‐parameters		
		Estimate	*SE*	*z*
Ocelot occupancy (*ψ*)				
GFC30	1.00	1.303	0.441	2.9566
SLO	0.58	−0.839	0.366	−2.2934
DCAD	0.51	0.542	0.332	1.6304
D.ROA	0.46	−2.426	0.921	−2.6355
D.RIV	0.42	−0.169	0.247	−0.6838
D.LAK	0.38	−0.959	0.624	−1.5372
ELE	0.37	−1.161	0.638	−1.8177
D.SET	0.30	0.013	0.416	0.0312
Ocelot detection (*p*)				
Effort	1.00	0.175	0.0289	6.050
PNCA	1.00	−4.563	0.3909	−11.671
PNM	1.00	1.620	0.2880	5.623
TMES	1.00	1.482	0.2924	5.067
RDSA	0.96	1.205	0.3027	3.982
Uatuma	0.90	−1.303	0.5024	−2.594
BRA319	0.89	−1.973	0.4003	−4.929
DUCKE	0.83	−1.143	0.5523	−2.070
PNJU	0.80	1.254	0.4668	2.687
REMJ & RSUA	0.74	1.032	0.3444	2.997
PBDFF	0.64	0.822	0.4718	1.743
SBR	0.46	−0.229	0.6086	−0.377
ZF2	0.36	0.252	0.4306	0.586

*Notes*

AIC_c_ Akaike's information criterion corrected for finite sample sizes. ΔAIC_c_ relative difference in AIC_c_ values compared with the top ranked model, AIC_c_wt weight, *K* number of parameters. Site covariates tested were: elevation (ELE), slope (SLO), distance to rivers (D.RIV), distance to lakes (D.LAK), distance to roads (D.ROA), distance to settlements (D.SET), Global Forest Change with threshold values 30 (GFC30) and disjunct core area density (DCAD). Detection covariates tested were as follows: effort and site. Estimates and standard error (*SE*) of untransformed covariate effects (*β* parameters) are given for the most parsimonious model that included the covariate.

### 3.3 Best model accounting for spatial autocorrelation

The posterior predictive loss criteria were slightly different for the model with the spatial correlation parameter (*D* = 485.1454) and without that parameter (*D* = 485.3477). In addition, the posterior variation was larger for the nonspatial model. Further, the posterior distribution of the spatial variance parameter (σ=1/√τ) was far from zero (95% credible interval of 8.4975–59039.02), implying that additional spatial correlation in the occupancy process strongly contributed to the variation in the habitat use probabilities. Based on the 95% credible intervals of the covariates, there was strong evidence to suggest that for both nonspatial models and spatial models, Global Forest Change Threshold 30% (GFC30) was significantly associated with habitat use as the 95% CI did not overlap zero, while slope (SLO) and DCAD were not significantly correlated with habitat use (Supporting Information Table S5).

**Figure 4:**
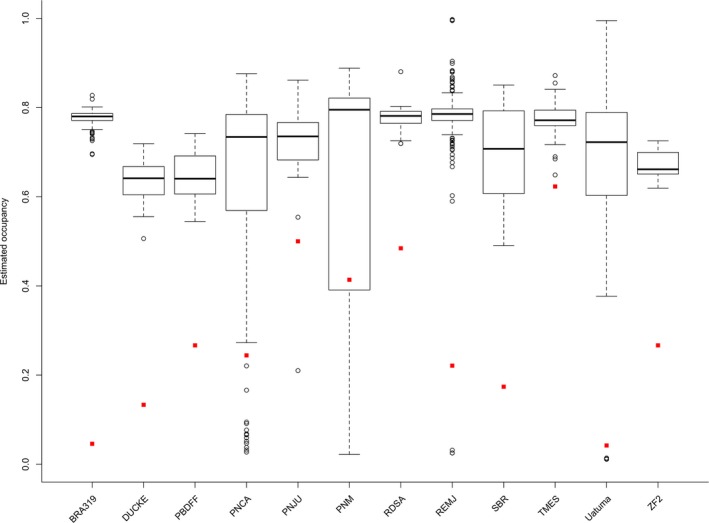
Boxplot shows estimated ocelot's occupancy incorporating spatial autocorrelation in each surveyed site, and the red dots were the naïve occupancy in each surveyed site

The protected area PNM had the highest estimated habitat use probability, followed by TMES and PNJU (Supporting Information Table S5). For all protected areas, the naïve habitat use probability was much lower than the estimated habitat use probability, showing evidence of ocelot imperfect detection (Figure 4). Compared to models not taking spatial autocorrelation into account, models incorporating spatial autocorrelation resulted in slightly lower occupancy estimates for the majority of surveyed areas (expect for DUCKE, PBDFF, PNJU, and ZF2; Table 4).

**Table 4:** Average probability of occupancy and standard error (*SE*) from spatial and nonspatial occupancy models, based on the model *p*(site + effort), *ψ*(GFC30 + DCAD + SLO)

	RSR models		Nonspatial models		Occupancy (%)
	Occupancy (%)	*SE*	Occupancy (%)	*SE*	
BRA319	77.71	0.4021	77.88	0.4021	4.59
PNCA	62.79	0.3043	62.97	0.3043	24.42
PNM	59.99	0.2060	60.42	0.2060	41.38
RDSA	79.59	0.2498	79.77	0.2498	48.44
REMJ&RUSA	76.61	0.3481	77.82	0.3481	22.12
DUCKE	63.82	0.4262	62.98	0.4262	13.33
PBDFF	63.96	0.3645	63.37	0.3645	26.67
ZF2	68.42	0.3633	67.80	0.3633	26.67
TMES	77.67	0.1848	77.94	0.1848	62.30
PNJU	68.39	0.2263	68.25	0.2263	50.00
Uatuma	67.96	0.4266	70.19	0.4266	4.21
SBR	69.43	0.3820	70.10	0.3820	17.39

*Notes*

Detection covariates were different surveyed area (site), and number of days a camera trap station was active for during each sampling occasion (effort). Occupancy covariates were Global Forest Change Threshold 30% (GFC30), disjunct core area density (DCAD), and slope (SLO). Restricted spatial regression (RSR) models incorporated spatial autocorrelation, while nonspatial models did not. Naïve occupancy estimate represented the estimate of occupancy obtained without incorporating variations in detection probability, occupancy covariates, or spatial autocorrelation.

## 4 DISCUSSION

We found that habitat use by ocelots is positively associated with forest cover, disjunct core area density, distance to roads/settlements, elevation, and negatively related to slope, distance to rivers/lakes. Nevertheless, ocelots also emerge as rather adaptable and their habitat use is not much influenced by other environmental variables. This suggests, as we would have predicted from their size and anatomy, that they are adaptable predators to a certain extent and are able to thrive wherever there are forests populated with suitable prey—a characterization that informs thinking about both their role as Neotropical carnivore guilds and their conservation.

Our results implied that the probability of habitat use by ocelots is various in different surveyed areas. The attribute of surveyed area might be one of the reasons to explain the variation of probability of habitat use in different study sites. The estimated probability of habitat use by ocelots in SBR was low because it was a private area, while other areas were protected area. This meant that the ocelot status is better in protected area than in private area. Another reason might be the human disturbance at a few of the protected areas. DUCKE, PBDFF, and ZF2 protected areas are fringed by city suburbs due to rapid urban expansion (Gonçalves, 2013). Our habitat use analysis revealed that the Global Forest Change threshold 30% (GFC30) had an important influence on ocelot occurrence: increased forest cover was associated with increased estimated probability of habitat use (sigmoidal relationship). This accords with findings from Peru and Texas, where ocelots preferred dense and closed canopy forest (Emmons, 1988; Haines, Grassman, Tewes, & Janečka, 2006). This was not unexpected insofar as greater forest cover was probably associated with higher prey availability (Droz & Pȩkalski, 2001, but see Hearn et al., 2017). Additionally, it has been suggested that the strong preference of ocelots for dense cover might also be related to the avoidance of potential competitors such as the bobcat (*Lynx rufus*) in South Texas (de Oliveira et al., 2010). It remains possible, however, that a positive relationship between ocelot habitat use and GFC30 arises because ocelots use less forested areas with lower probability. Although no longer statistically significant when spatial autocorrelation was taken into account, slope and disjunct core areas density (DCAD) were also influential covariates for ocelot habitat use. There are previous hints that the ocelot might avoid steeper slopes due to lower availability of prey there (de Oliveira et al., 2010). The positive relationship between DCAD and habitat use suggests that forest fragmentation process in some degree is favorable for ocelots concerning higher density of disconnected patches of suitable interior forest habitat, which supported by previous study about clouded leopard (*Neofelis nebulosi*; Tan et al., 2017).

All other covariates (importance <0.5) were not included in subsequent spatial autocorrelation analysis; however, they cannot ignore the influence on habitat use by ocelot. Our findings suggest that distance to road (D.ROA) emerged as important. Ocelots have been recorded killed on roads in the Tariquía−Baritú corridor between Bolivia and Argentina (Cuyckens, Falke, & Petracca, 2014). Similarly, we found that distance to settlements had a negative effect, although this was weak (importance = 0.30). Distance to roads and settlements may be to do with persecution by/avoidance of humans or indirect anthropogenic impacts like overhunting of prey. Temporal avoidance of ocelot in the presence of humans (Massara, de Oliveira Paschoal et al., 2018; Massara, Paschoal et al., 2018; Pardo Vargas, Cove, Spinola, de la Cruz, & Saenz, 2016) and other competitor, puma (Massara, de Oliveira Paschoal et al., 2018; Massara, Paschoal et al., 2018) has been observed, which also suggests that ocelots might avoid human activities or other larger species. As predicted, elevation was also influential covariate for ocelot habitat use. Previous studies indicated that the probability of habitat use by ocelots decreased with elevation (Ahumada, Hurtado, & Lizcano, 2013; Di Bitetti, Albanesi, Foguet, De Angelo, & Brown, 2013). Perhaps this is because lowland forests have higher net primary productivity (Robertson et al., 2010), which may increase resources (Peres, 1994) to sustain a greater abundance of ocelot prey. These prey may, in a seasonal way, use lowland forests to take advantage of the abundant trophic resource in this forest type following the receding waters (Costa et al., 2018). However, it is important to note that variability in elevation throughout central and southern Brazilian Amazon extends over a limited range (22.56–241.34 m a.s.l., average 96.92 m), which might be one reason why the effect of elevation was weak (importance = 0.37). Distance to river/lakes was also omitted from our final model, but a previous study revealed that ocelots tend to aggregate near major rivers (Emmons, 1987). In our classification, water bodies included only major rivers and lakes so further analysis might need to focus on smaller streams and rivers deeper within protected area, because in the case of many areas in the Amazon that have great extensions of nonfloodable *terra firme* (dry land/solid ground), density of small streams may have influence. In addition, in our case, the camera trap stations were mainly concentrated at close proximity to rivers so further analysis should investigate whether the effect of river on ocelot occupancy still exist when considering further distances from rivers.

The presence of sympatric species can influence ocelot's habitat use in Atlantic Forest remnants: The presence of a top predator (jaguars, *P. onca*, and pumas, *P. concolor*) was positively associated with ocelot habitat use (Massara, de Oliveira Paschoal et al., 2018; Massara, Paschoal et al., 2018, Supporting Information Table S1). There was also a weaker negative relationship reported between numbers of domestic dogs (*Canis familiaris*) detected and ocelot occupancy (Massara, de Oliveira Paschoal et al., 2018; Massara, Paschoal et al., 2018, Supporting Information Table S1). This factor and the availability of prey or presence of apex predators were not included in our analysis. The prey of ocelots is mainly comprised of small and medium‐sized mammals such as the three‐toed sloth (*Bradypus variegatus*) and nine‐banded long‐nosed armadillo (*Dasypus novemcinctus*) but also includes birds, fish, and snakes (Emmons, 1987; Wang, 2002). The presence–absence of prey might be a key and more immediate factor than forest cover or water availability in explaining ocelot habitat use pattern. There are some studies focused on the sympatric species or prey of ocelot (Massara, de Oliveira Paschoal et al., 2018; Massara et al., 2016; Massara, Paschoal et al., 2018; Pratas‐Santiago et al., 2016; Supporting Information Table S1), in the future they could be studied using multispecies occupancy models (Rota et al., 2016) and piecewise structural equation modeling (SEM; Geary, Ritchie, Lawton, Healey, & Nimmo, 2018; Grace et al., 2012).

Our results prompt comparisons with other similar mesopredators, such as the clouded leopard, which would appear to be an ecological analog of the ocelot. They have some commonalities, such as similar size (11–23 kg for clouded leopard), activity pattern (Di Bitetti et al., 2006; Grassman, Tewes, Silvy, & Kreetiyutanont, 2005), and similar functional role in the ecosystem. A study in Peninsular Malaysia indicated that clouded leopard habitat use increased with increasing distance to rivers or streams and higher elevation. Our findings for ocelot mirrored this elevation effect, but not the effect of distance to rivers. Furthermore, DCAD was a strong contributory factor for ocelots, and similarly it was a positive influence on habitat use of the Malaysian clouded leopard (Tan et al., 2017). Tan et al. (2017) also found that habitat use by clouded leopards was positively associated with forest cover, mirroring our results for ocelots. Findings like these start to resolve the niche differentiation of these seemingly similar felids which co‐occur and share an evolutionary history. Nevertheless, in general, forest cover, topographical factor (elevation or slope), distance to water (river or lakes), and distance to roads and settlements all emerge as important to these medium‐sized felids.

Unsurprisingly, the results indicate that detection probability was positively correlated with camera trapping efforts and was not constant across all survey areas. This was to be expected because the longer a camera trap survey, the higher the probability of detecting a species. In fact, the increase in the sample efforts to obtain more robust data should be encouraged, leaving cameras for at least 90–120 days in the field and having several years of sampling. Meanwhile, there are other factors that might lead to different detection probabilities, such as seasonality (period of the year that the surveys were carried out: e.g., dry or rainy season) and the number of camera traps at a station (paired or single cameras). The two sites (REMJ & RSUA and RDSA) with different detection probabilities that are situated in the far west of Brazilian Amazonia illustrate strong influences of rivers and seasonal flooding (see also Costa et al., 2018). Previous reports revealed that position of camera trap stations (on trails or not) might also affect the detection probability: The detection probability was higher for camera trap stations located on roads than on trails (Di Bitetti et al., 2006). Additionally, variation in ocelot density at different surveyed areas will also affect detectability, with a higher ocelot density associated with higher detectability (Massara, De Oliveira Paschoal, Doherty, Hirsch, & Chiarello, 2015). Many sources of evidence point to a gradient in productivity and biomass, being higher in the western/south western Amazon and lower in the central and eastern parts of the basin (Houghton, Lawrence, Hackler, & Brown, 2001). This gradient could influence density and abundance, therefore, detection probability. In our study, we accounted for spatial autocorrelation (Johnson et al., 2013) to obtain a more accurate estimate for ocelot habitat use. This correction is biologically important (Poley et al., 2014) but often neglected (Hodges & Reich, 2010).

However, a limitation of our study is that all our surveys were conducted in prime habitat (except SBR and part of BRA319). Within the range of variation we studied, ocelots were ubiquitous. A further limitation is that we did not consider prey and sympatric predators. Our findings that ocelots were ubiquitous, and seemingly abundant in protected areas, do not justify complacency regarding their conservation: Deforestation is destroying their habitat. Ocelots are strong candidates for conservation ambassador species (Macdonald et al., 2017), so their conservation transcends benefits to their own populations, but extends to the species with which they are sympatric, and the habitats they occupy.

## CONFLICT OF INTEREST

None declared.

## AUTHORS’ CONTRIBUTIONS

CKWT and DGR conceived the idea for this article. Data acquisition was performed by DGR, MIA, APA, HCMC, ALSG, MJDP, JP, ER, MLR, and ECJ. Data analysis was primarily conducted by BXW with help from CKWT and DGR. The article was primarily written by BXW. All authors contributed critically to the drafts and gave final approval for publication.

## Data Availability

Data and sampling locations available through the Dryad https://doi.org/10.5061/dryad.p7410jm.
