# Supplementary material for: Habitat use of the ocelot (Leopardus pardalis) in Brazilian Amazon
Source: Ecol Evol. 2019 Apr 19;9(9):5049–62. doi: 10.1002/ece3.5005 (PMC6509378; doi:10.1002/ece3.5005)
Supplement: Supplementary file 1 [file ECE3-9-5049-s001.doc]

Appendix

Table S1

Comparison of ocelot studies using camera traps

| Studies | Year | Region | Research questions | Main findings |
| --- | --- | --- | --- | --- |
| Massara et al | 2018 | Brazilian Atlantic Forest reserves | Factors influencing ocelot occupancy | Positive correlation between occupancy of ocelots and top predators; ocelot’s local distribution is influenced by domestic dogs |
| Huck et al | 2017 | Northern Argentina | Activity patterns | Ocelots have a slight tendency for higher activity during brighter nights. |
| Massara et al | 2016 | Southeastern Brazil | The influence on the other 6 mesocarnivores from ocelot | Ocelot occurrence did not influence habitat use of these mesocarnivores |
| Porfirio et al | 2016 | Brazilian Pantanal | Activity patterns | Ocelots may tailor their activity to that of some of their potential prey |
| Pratas-Santiago et al | 2016 | Central Amazonia | Effect of the moon cycle on this predator-prey relationship | Ocelots switch type of prey (diurnal or nocturnal) they hunt in different moon phases |
| Salvador et al | 2016 | Yasuni National Park, Ecuador | Abundance | 0.31 (SE +/- 6) to 0.85 (SE +/- 17) ocelots/km^2^ in Maxus Road  0.35 (SE +/- 6) to 0.93 (SE +/- 18) ocelots/km^2^ in Lorocachi |
| da Rocha et al | 2016 | Central Amazonia | Density | 24.84 +/- SE 6.27 ocelots per 100 km^2^ |
| Blake et al | 2016 | Eastern Ecuador | Activity patterns | Males and females showed similar hourly patterns of activity (nocturnal). |
| Penido et al | 2016 | Northeastern Brazil | Density | 3.16 +/- 0.46 individuals per 100 km^2^(secr) |
| Massara et al | 2015 | Protected Brazilian Atlantic Forest | Abundance | Ocelots respond negatively to habitat loss |
| Martinez et al | 2015 | Sierra Abra-Tanchipa Biosphere Reserve | Density | 0.04(dry seasons) , 0.03-0.18(humid seasons) individuals/ km2 |
| Rodgers et al | 2015 | Barro Colorado Island | Whether communal latrines act as important role | Communal latrines are important centres of scent communication |
| Rodgers et al | 2015 | Barro Colorado Island | Kin selection plays a role in structuring ocelot populations? | Kin selection influences ocelot spatial organization |
| Perez-Irineo et al | 2014 | Southeast Mexican rainforests | Density, activity pattern, sex ratio | 22-38 individuals/100km2 ;sex ratio was statistically equal to 1:1; Ocelot activity was more frequent at night (1:00-6:00h) |
| Aranda et al | 2014 | Lagunas de Zempoala National Park | First record | New record at the highest altitude |
| Emsens et al | 2014 | Barro Colorado Island | Whether prey refuges attract predators | Ocelots can be attracted to prey refuges or refuging prey |

| Cove et al | 2014 | San Juan - La Selva Biological Corridor, Costa Rica | Effectiveness of common visual and olfactory attractants in field settings | Employing several attractants as the most appropriate way to survey ocelots |
| --- | --- | --- | --- | --- |
| Rodgers et al | 2014 | Barro Colorado Island | Density | 1.74/km^2^ from non-invasive genetics, 1.59/km^2^ from camera trapping |
| Ahumada et al | 2013 | Northern Jalisco, Mexico | New record | High-elevation records of ocelot |
| Moreno et al | 2011 | Jalisco, Mexico | New record | High-elevation records of ocelot |
| Manuel et al | 2011 | San Luis Potosi, Mexico | Distribution | Ocelot range is more extended to the west than its original geographical range |
| Gonzalez et al | 2011 | The Talamanca region, Costa Rica | Density | 8.95, 10.33 and 11.61 individuals (Mo model) and 5.59, 6.45 and 7.25 (Mh model) individuals x 100 km^-2^ |
| Kolowski et al | 2010 | Northern Peru | Density and activity patterns | 75.2 ocelots/100km^2^(control),94.7 ocelots/100km^2^(disturbance period) |
| Fusco et al | 2010 | Atlantic Forest, Brazil | Density | 0.21±0.03 individuals/km2 |
| Iglesias et al | 2008 | Guanajuato, Mexico | New record | New record of ocelot |
| Dillon et al | 2008 | Central America | Home range, overlap and density | 26.09 km^2^ (95% fixed kernel) and 18.91 km^2^ (100% minimum convex polygon);12.61 ocelots per 100 km^2^ |
| Di Bitetti et al | 2008 | Atlantic Forest of Misiones, Argentina | Ocelot abundance | 4.96 +/- 1.33 individuals per 100 km^2^in the logged and hunted areas to 17.6 +/- 2.25 individuals per 100 km^2^ in areas with low human impact. |
| Maffei et al | 2008 | Bolivian chaco | Density | Sampling areas estimated with camera traps to assess ocelot density are reliable |
| Dillon et al | 2007 | Belize | Density | 5.82-25.88 per 100 km^2^ in the broad-leaf versus 2.31-3.80 per 100 km^2^ |
| Di Bitetti et al | 2006 | Atlantic Forest of Misiones, Argentina | Density, habitat use and activity patterns | 7.7 +/- 1.4 to 13.4 +/- 2.6 individuals x 100 km^-2^ |
| Haines et al | 2006 | Southern Texas, United States | Importance of private landowners in the ocelot recovery process | 0.30 +/- SE 0.03 ocelots km^-2^ |
| Maffei et al | 2005 | Eastern Bolivia | Densities, activity, and ranging behaviour | Average population density of 0.3 ocelots km^−2^ |
| Trolle et al | 2005 | Northern Pantanal | Abundance | 0.112 independent individuals/km^2^ (SE 0.069) |
| Trolle et al | 2003 | Brazilian Pantanal | Density | 2.82 independent individuals/every 5 km^2^ (SE 1.00) |

Table S2

Chi-square probability (X^2^p) and over dispersion statistic (ĉ) results of the MacKenzie and Bailey (2004) goodness of fit test for ocelot occupancy models with different collapsing day-periods in Central Amazon. Detection covariates were number of days a camera trap station was active for each sampling occasion (EFFORT). Occupancy covariates were elevation (ELE), slope (SLO), distance to rivers (D.RIV), and distance to lakes (D.LAK), distance to roads (D.ROA), distance to settlements (D.SET), Vegetation Continuous Field (VCF), Global Forest Change with four different threshold values (GFC30, GFC50, GFC75, GFC90), Disjunct Core Area Density(DCAD), Contrast-weighted edge density(CWED), Contiguity index (Contig). The dataset was limited to 120 sampling days and total number of camera-trap station was 899.

| Model | X^2^p | ĉ |
| --- | --- | --- |
| 7-days sampling occasions |  |  |
| p(EFFORT+site), psi(ELE+SLO+D.RIV+D.ROA+D.LAK+D.SET+VCF+GCF+CWED+Contig+DCAD) | 0.22 | 0.3846 |
| 10-days sampling occasions |  |  |
| p(EFFORT+site), psi(ELE+SLO+D.RIV+D.ROA+D.LAK+D.SET+VCF+GCF+CWED+Contig+DCAD) | 0.45 | 0.5206 |
| 12-days sampling occasions |  |  |
| p(EFFORT+site), psi(ELE+SLO+D.RIV+D.ROA+D.LAK+D.SET+VCF+GCF+CWED+Contig+DCAD) | 0.25 | 0.6662 |
| 15-days sampling occasions |  |  |
| p(EFFORT+site), psi(ELE+SLO+D.RIV+D.ROA+D.LAK+D.SET+VCF+GCF+CWED+Contig+DCAD) | 0.36 | 0.6222 |

Table S3

Summary of site covariates (mean+SD). Occupancy covariates were elevation (ELE), slope (SLO), distance to river (D.RIV), distance to roads (D.ROA), distance to lakes (D.LAK), distance to settlements (D.SET), Vegetation Continuous Field (VCF), Global Forest Change with four different threshold values (GFC30, GFC50, GFC75, GFC90), Contiguity index (Contig), Contrast-weighted edge density (CWED) and Disjunct core area density (DCAD).

| Covariates | BRA319 | DUCKE | PBDFF | PNCA | PNJU | PNM | RDSA | REMJ | SBR | TMES | Uatuma | ZF2 |
| --- | --- | --- | --- | --- | --- | --- | --- | --- | --- | --- | --- | --- |
| ELE | 58.76±8.55 | 94.08±11.58 | 117.85±17.51 | 72.80±19.94 | 209.65±11.29 | 98.49±25.71 | 116.92±17.43 | 104.56±10.93 | 199.51±20.81 | 175.25±7.71 | 65.29±23.08 | 93.17±17.58 |
| SLO | 2.46±0.57 | 6.78±1.24 | 6.62±1.60 | 3.83±2.34 | 4.28±1.34 | 2.97±1.97 | 2.56±0.86 | 2.79±1.12 | 4.09±1.08 | 3.55±0.94 | 5.45±2.98 | 5.77±0.84 |
| D.ROA | 1.82±1.44 | 4.24±1.51 | 8.43±5.39 | 51.31±11.35 | 67.76±4.62 | 34.58±11.40 | 241.31±3.72 | 319.04±198.78 | 51.05±2.09 | 104.30±5.42 | 363.69±20.35 | 121.17±6.71 |
| D.RIV | 1.26±0.99 | 0.77±0.71 | 0.49±0.60 | 1.16±0.93 | 0.65±0.58 | 2.81±1.76 | 1.37±1.12 | 1.07±0.95 | 0.5±0.5 | 1.31±1.19 | 1.72±1.21 | 1.65±1.05 |
| D.LAK | 39.77±9.55 | 11.23±1.87 | 50.09±2.80 | 38.81±26.59 | 2.27±1.46 | 37.56±14.26 | 6.53±3.28 | 5.23±3.86 | 33.67±2.13 | 7.87±4.98 | 2.84±2.63 | 18.55±6.86 |
| D.SET | 67.95±28.93 | 26.52±1.59 | 30.01±3.37 | 158.55±195.55 | 244.59±2.32 | 87.72±44.53 | 50.35±3.92 | 39.30±28.96 | 227.15±2.00 | 360.52±3.20 | 36.69±22.31 | 13.11±6.74 |
| GFC30 | 1.00±0.01 | 1.00±0 | 1.00±0 | 0.84±0.30 | 0.95±0.14 | 0.79±0.35 | 1±0.02 | 1±0.00 | 0.90±0.10 | 1.00±0.01 | 0.99±0.05 | 1.00±0 |
| GFC50 | 1.00±0.01 | 1.00±0 | 1.00±0 | 0.78±0.36 | 0.95±0.15 | 0.76±0.38 | 1±0.03 | 1±0.01 | 0.88±012 | 1.00±0.01 | 0.99±0.05 | 1.00±0 |
| GFC75 | 1.00±0.01 | 1.00±0 | 1.00±0 | 0.74±0.39 | 0.94±0.15 | 0.72±0.40 | 1±0.03 | 1±0.01 | 0.88±0.12 | 1.00±0.01 | 0.99±0.06 | 1.00±0 |
| GFC90 | 1.00±0.02 | 1.00±0 | 1.00±0 | 0.72±0.40 | 0.94±0.15 | 0.71±0.40 | 1±0.03 | 1±0.01 | 0.85±0.12 | 0.99±0.01 | 0.99±0.06 | 1.00±0 |
| VCF | 80.36±2.31 | 71.50±6.98 | 79.71±4.52 | 59.26±25.31 | 78.98±5.21 | 54.89±22.06 | 79.13±2.37 | 77.30±5.46 | 78.63±16.04 | 68.14±6.67 | 71.92±10.45 | 77.30±3.14 |
| Contig | 0.93±0.10 | 0.91±0.12 | 0.85±0.14 | 0.60±0.23 | 0.80±0.23 | 0.52±0.18 | 0.92±0.13 | 0.81±0.20 | 0.52±0.21 | 0.59±0.23 | 0.73±0.23 | 0.92±0.07 |
| CWED | 0.65±3.44 | 0.23±0.52 | 0.51±0.80 | 20.69±20.98 | 6.42±13.65 | 24.70±16.44 | 2.06±9.20 | 6.90±12.50 | 30.08±22.71 | 5.54±7.70 | 12.60±18.45 | 0.25±0.58 |
| DCAD | 1.65±1.35 | 1,56±0.49 | 1.76±0.59 | 4.71±3.54 | 3.67±4.82 | 7.05±4.95 | 2.12±3.45 | 3.39±4.08 | 9.02±6.78 | 4.66±3.76 | 5.14±5.34 | 1.49±0.25 |

Table S4

Ocelot single covariate occupancy (ψ) models. AICc akaike’s information criterion corrected for finite sample sizes. ΔAICc relative difference in AICc values compared with the top ranked model, AICcwt weight, K number of parameters. Site covariates tested were: elevation (ELE), slope (SLO), distance to rivers (D.RIV), distance to lakes (D.LAK), distance to roads (D.ROA), distance to settlements (D.SET), Vegetation Continuous Field (VCF), Global Forest Change with four different threshold values (GFC30, GFC50, GFC75, GFC90), Disjunct Core Area Density (DCAD), Contrast-weighted edge density (CWED) and Contiguity index (Contig).

|  | AICc | ΔAICc | AICcWt | K | log likelihood |
| --- | --- | --- | --- | --- | --- |
| *ψ*(GFC30) | 1767.78 | 0.00 | 0.73 | 15 | -868.62 |
| *ψ*(GFC50) | 1771.07 | 3.30 | 0.14 | 15 | -870.26 |
| *ψ*(GFC75) | 1772.53 | 4.75 | 0.07 | 15 | -870.99 |
| *ψ*(GFC90) | 1772.69 | 4.91 | 0.06 | 15 | -871.07 |
| *ψ*(VCF) | 1782.68 | 14.90 | 0.00 | 15 | -876.07 |
| *ψ*(D.LAK) | 1785.61 | 17.83 | 0.00 | 15 | -877.53 |
| *ψ*(D.ROA) | 1789.68 | 21.90 | 0.00 | 15 | -879.57 |
| *ψ*(D.RIV) | 1789.76 | 21.98 | 0.00 | 15 | -879.61 |
| *ψ*(.) | 1790.44 | 22.67 | 0.00 | 14 | -880.98 |
| *ψ*(DCAD) | 1791.09 | 23.31 | 0.00 | 15 | -880.27 |
| *ψ*(ELE) | 1791.12 | 23.34 | 0.00 | 15 | -880.29 |
| *Ψ*(SET) | 1791.49 | 23.71 | 0.00 | 15 | -880.47 |
| *Ψ*(CWED) | 1791.90 | 24.12 | 0.00 | 15 | -880.68 |
| *ψ*(SLO) | 1792.35 | 24.58 | 0.00 | 15 | -880.91 |
| *ψ*(Contig) | 1792.46 | 24.68 | 0.00 | 15 | -880.96 |

Table S5

Covariate coefficients and credible interval (CI) based on 2.5% and 97.5% quartiles, produced by models with and without accounting for spatial autocorrelation. Occupancy covariates tested were: Global Forest Change Threshold 30% (GFC30), slope (SLO) and disjunct core area density (DCAD). Bold CI indicate that zero is not within interval.

|  | Without spatial autocorrelation | | | With spatial autocorrelation | | |
| --- | --- | --- | --- | --- | --- | --- |
|  | Mean | SD | 95% CI | Mean | SD | 95% CI |
| *p* |  |  |  |  |  |  |
| (Intercept) | -2.364 | 0.193 | **(-2.747, -1.990)** | -2.363 | 0.194 | **(-2.749, -1.990)** |
| PNM | 0.933 | 0.178 | **(0.592, 1.290)** | 0.937 | 0.176 | **(0.598,1.284)** |
| RDSA | 0.461 | 0.157 | **(0.153, 0.770)** | 0.459 | 0.155 | **(0.156, 0.765)** |
| REMJ&RSUA | 0.144 | 0.139 | (-0.129, 0.417) | 0.150 | 0.139 | (-0.121, 0.423) |
| PNJU | 0.555 | 0.225 | **(0.120, 0.997)** | 0.549 | 0.225 | **(0.115, 0.994)** |
| SBR | -0.326 | 0.253 | (-0.833, 0.163) | -0.326 | 0.254 | (-0.838, 0.164) |
| Uatuma | -0.550 | 0.224 | **(-1.007, -0.127)** | -0.540 | 0.225 | **(-0.996, -0.111)** |
| TMES | 0.727 | 0.147 | **(0.439, 1.017)** | 0.728 | 0.146 | **(0.442, 1.015)** |
| DUCKE | -0.460 | 0.277 | (-1.013, 0.076) | -0.468 | 0.277 | (-1.020, 0.071) |
| ZF2 | 0.214 | 0.251 | (-0.277, 0.0.710) | 0.202 | 0.250 | (-0.284, 0.696) |
| PBDFF | 0.491 | 0.268 | (-0.013, 1.045) | 0.480 | 0.267 | (-0.023, 1.029) |
| BRA319 | -0.873 | 0.174 | **(-1.221, -0.536)** | -0.873 | 0.174 | **(-1.218, -0.536)** |
| Effort | 0.090 | 0.014 | **(0.062, 0.119)** | 0.090 | 0.014 | **(0.062, 0.119)** |
| *ψ* |  |  |  |  |  |  |
| (Intercept) | 0.6582 | 0.2621 | **(0.289, 1.330)** | 0.649 | 0.249 | **(0.281, 1.226)** |
| GFC30 | 0.4854 | 0.1143 | **(0.285, 0.732)** | 0.478 | 0.111 | **(0.282, 0.715)** |
| SLO | -0.2109 | 0.1793 | (-0.522, 0.177) | -0.190 | 0.198 | (-0.498, 0.251) |
| DCAD | 0.1732 | 0.1582 | (-0.081, 0.546) | 0.153 | 0.149 | (-0.090, 0.503) |

Table S6

Model selection statistics for eight models fit to ocelot data. AICc akaike’s information criterion corrected for finite sample sizes. ΔAICc relative difference in AICc values compared with the top ranked model, AICcwt weight, K number of parameters.

|  | AIC_c_ | ΔAIC_c_ | AIC_c_wt | K | -2*loglikelihood |
| --- | --- | --- | --- | --- | --- |
| ψ(.), *p*(site+effort) | 1789.83 | 0.00 | 0.8538 | 14 | 1761.83 |
| ψ(.),θ(.),θ(.)’, *p*(site+effort),θ(.)pi | 1793.36 | 3.53 | 0.1462 | 17 | 1759.36 |
| ψ(.), *p*(site) | 1835.14 | 45.31 | 0 | 13 | 1808.59 |
| ψ(.),θ(.),θ(.)’, *p*(site),θ(.)pi | 1838.23 | 48.40 | 0 | 16 | 1806.23 |
| ψ(.), *p*(effort) | 1945.91 | 156.08 | 0 | 3 | 1939.33 |
| ψ(.),θ(.),θ(.)’, *p*(effort)θ(.)pi | 1967.33 | 177.50 | 0 | 5 | 1957.33 |
| ψ(.), *p*(.) | 1985.91 | 196.08 | 0 | 2 | 1981.90 |
| ψ(.),θ(.),θ(.)’, *p*(.)θ(.)pi | 1985.96 | 196.13 | 0 | 5 | 1975.96 |
